# Graphene Oxide Incorporated Nanohybrid Aerogels as New Generation Drug Carrier Platforms

**DOI:** 10.3390/gels11120949

**Published:** 2025-11-26

**Authors:** Elif Çalışkan Salihi, Shalaleh Hasan Niari Niar, Elif Nur Ulucici, Ebrar Tuğba Gedik, Fatma Betül Zengin, Fulya Samur, Seda Didem Şahin, Turgut Taşkın

**Affiliations:** 1 Department of Basic Pharmaceutical Sciences, Faculty of Pharmacy, Marmara University, Istanbul 34854, Türkiye; fulyasamurr@gmail.com (F.S.); sedadidemsahinn@gmail.com (S.D.Ş.); 2 ECS Graphene R&D Engineering and Consultancy, Marmara University Technopark, Istanbul 34854, Türkiye; 3 Department of Basic Pharmaceutical Sciences, Institute of Health Sciences, Marmara University, Istanbul 34865, Türkiye; shalale.hniariniar@gmail.com (S.H.N.N.); elifnurtolu@hotmail.com (E.N.U.); ebtgedik@gmail.com (E.T.G.); 4 Department of Pharmacognosy, Faculty of Pharmacy, Marmara University, Istanbul 34854, Türkiye; fatma.zengin@marmara.edu.tr (F.B.Z.); ttaskin237@gmail.com (T.T.); 5 Department of Pharmacognosy, Institute of Health Sciences, Marmara University, Istanbul 34865, Türkiye

**Keywords:** graphene oxide, nanohybrid, aerogel, diclofenac sodium, adsorption

## Abstract

By combining the superior capacity of graphene oxide with alginate matrix in a nanohybrid aerogel structure, great potential can be developed for technical features and use in areas such as biomedicine. The aim of this study is the production of graphene oxide incorporated nanohybrid aerogels using ambient pressure drying technique for drug loading and release. The produced nanostructures were identified with electron microscopy, X-ray diffraction, dynamic light scattering, and spectroscopic techniques. The surface area of the nanomaterials was examined by methylene blue method. Drug adsorption capacity of the nanohybrid aerogels was investigated by conducting batch adsorption studies using the model drug diclofenac sodium (DS). The equilibrium time of drug adsorption was determined and adsorption kinetics were modeled. The adsorption equilibrium time of the drug on the adsorbent was found to be 3 h. Equilibrium data were modeled by using the Langmuir and the Freundlich isotherms and according to the Langmuir isotherm, the maximum adsorption capacity was found to be 20.83 mg/g. Results showed the potential of the produced nanohybrid aerogels to be used as drug adsorbents for drug delivery applications. The results obtained from this study will be useful in drug production and treatment.

## 1. Introduction

Graphene is a two-dimensional material consisting of sp^2^ hybridized carbon atoms, with a honeycomb and lattice structure. It has various forms according to dimensional differences. Graphene quantum dots are zero-dimensional; pure graphene, graphene oxide, graphene, and reduced graphene oxide are two-dimensional structures; graphene foam and graphene aerogel forms are three-dimensional forms. In this nano-sized carbon material, carbons are bonded to each other by 0.142 nm long Van Der Waals bonds [1,2,3,4,5]. Historically, when we look at its production, it was first synthesized in graphene oxide form by the Brodie method [6]. With the Nobel Prize in Physics in 2010, graphene production and research have become widespread; graphene has been functionalized with various agents. With the help of hydrogen bonds, covalent bonds, and many other bonds, graphene and graphene-based materials have been produced from past to present using different techniques [7].

The unique physicochemical properties and high biocompatibility of graphene and graphene-based materials have made them stand out in biomaterial applications. In this way, graphene-based materials are used in many areas from pharmacy [8] to industry [9,10], from tissue engineering [11] to energy technologies [12]. The use of graphene-based materials in drug delivery system applications, especially in the field of pharmacy, is one of the important current issues. New and effective drug carrier materials are needed to improve the therapeutic effects of drugs to be used in targeted therapy. In the use of traditional drug delivery systems, many problems have been encountered, such as the rapid release of the active substance, while the therapeutic effects of drugs decrease. Targeted therapy has been developed in response to these problems aims to ensure that the drug used reaches the desired tissue effectively and thus reduce the side effects of the drug, thereby increasing the quality of life of the patients and the effectiveness of the treatment, and providing fast and effective results without using high doses of the drug [13,14,15]. Depending on the viscosity of the medium, the carrier substance should have the character of delivering the drug to the target without leaking into healthy tissues [16]. Examples of nanocarriers include liposomes, dendrimers, and carbon-based materials. In addition to their disadvantages such as cytotoxicity, lack of long-term biocompatibility analysis, problems in large-scale synthesis, and inadequacy in animal studies, they can be advantageous in drug delivery systems with their positive responses to stimuli such as temperature, pH, redox, solvent type interacted with in the target tissue, electric field, and magnetic field [17,18,19,20,21]. In addition, the structural tunability of these different nanostructured materials provides targeted drug delivery with lower potential side effects. At this point, graphene comes to the forefront [22].

The multifunctional structure and large surface area of graphene make it suitable for use as a drug carrier material that will provide the specified effects. Results such as cell surface penetration and hydrophilicity due to its improved compatible structure and high electrostatic effect can be achieved with graphene [23,24,25]. Spectroscopic analyses revealed significant performance improvements during the drug loading phase of graphene functionalization. The resulting surface structure, which is more transparent and smooth, is among the positive aspects of functionalization, contributing to a more effective first step toward achieving targeted sites [25]. Especially in combined therapies, which involve combining the drug, graphene is seen as a good agent [26]. In addition to these positive properties, it has been observed in studies that the distance between the layers forming graphene is shortened by stacking them on top of each other and that there is a decrease in the large surface area specific to graphene [27]. At this point, the pore structure, which is an alternative drug loading option, emerges. The controllable pores and stable three-dimensional structures of graphene-based aerogels are thought to be an alternative to the potential stacking problems.

Alginate stands out as an environmentally friendly, non-toxic, effective, and economical material derived from biodegradable and renewable natural resources. It can swell in aqueous media to form a gel structure with ionic cross-links and is easily degraded by microorganisms [28,29]. Therefore, alginate is considered a prominent component in sustainable biomaterial design. These properties of alginate increase the biodegradability of composite systems formed with graphene oxide (GO) and provide environmental advantages [30,31]. GO is not only safer from an environmental perspective but also may exhibit more favorable biocompatibility properties. Although not directly biodegradable [32], it has been shown that GO can be degraded by some oxidative enzymes (e.g., peroxidases) in biological environments and converted into smaller carbon-based derivatives [33]. This biodegradation process reduces the environmental persistence of GO-containing materials and makes their interactions with living systems more controlled. However, in environmental systems, especially in aqueous systems, GO also undergoes different physicochemical processes such as redox reactions [34], sedimentation [35], and photodegradation [36]. Furthermore, under high pH conditions, deprotonation of ionizable functional groups on the surface can increase the interaction of GO with natural colloids, leading to ligand binding, aggregation, and ultimately sedimentation in water. This reduces the mobility of GO and is considered a potential environmental risk because it can cause it to accumulate in aquatic environments [37,38]. 

The GO–alginate based aerogels developed in this study are inherently recyclable and environmentally compatible due to their components. Alginate, thanks to its solubility, can be chemically reprocessed and converted back into a gel structure, while GO can be refunctionalized under physical and chemical conditions. The dried form of the aerogel can be ground and reused or reformulated in different systems. This demonstrates that the composites we developed can be considered not only for single-use but also multi-purpose recyclable platforms. In conclusion, thanks to the natural polymers and controlled GO ratios used, these composite aerogels offer a biocompatible, biodegradable, and recyclable drug delivery system with a low environmental footprint. These features reveal that our work can be evaluated not only in pharmaceutical applications but also in green technologies developed in line with the principles of sustainable material design and circular economy.

Aerogels are stable, three-dimensional, low-density, and easily functionalizable structures used in nano- to macro-scale studies with their porous and large surface areas. The highly porous structures of aerogels provide great advantages in many stages from drug loading to controlled drug transport and release to the target point. Three-dimensional graphene aerogel structures can be produced by combining aerogels with graphene and its derivatives such as GO, reduced GO (rGO), and other two-dimensional matrix materials. With this production, significant results can be obtained in targeted drug delivery by using the enhanced adsorption advantage of both graphene and aerogels [39,40,41]. In this study, within the framework of these potential positive effects, it was aimed to produce graphene oxide incorporated aerogels for pharmaceutical applications and to investigate the drug loading capacity level of this produced structure. For this purpose, the produced aerogels were characterized and their drug loading capacities were tested by adsorption studies. Diclofenac sodium (DS), a non-steroidal anti-inflammatory drug, was selected as a model drug for adsorption. The adsorption of DS on hybrid aerogels was investigated using different times and drug concentrations. Then, adsorption experiments were conducted and adsorption kinetics and equilibrium data were modeled.

Aerogels were chosen over hydrogels because one of the most important structural features of aerogels is that the gas phase within their pores is much more pronounced than the liquid phase within hydrogel networks. This large number of pores with adjustable sizes provides aerogels with a large surface area and makes them uniquely suited for drug loading and delivery. Furthermore, hydrogels offer significant advantages in topical applications, particularly due to their biocompatibility, flexibility, and sensitivity to environmental stimuli. Aerogels, on the other hand, stand out for their high porosity and mechanical strength, enabling rapid and effective drug release in various dosage forms. Consequently, the selection of hydrogels and aerogels should be made optimally depending on the target application area, drug type, and conditions of use [42,43].

Diclofenac sodium, a non-steroidal anti-inflammatory drug that is poorly soluble in water, is used in the treatment of diseases that develop inflammation, such as chronic muscle pain, pancreatitis, and rheumatoid arthritis. It reduces prostaglandin synthesis as a result of cyclooxygenase inhibition. This is the basic mechanism of its anti-inflammatory effect. On the other hand, cyclooxygenase enzyme inhibition causes various side effects [44]. When we look at its consumption, inflammatory diseases have become widespread with the increase in the elderly population in the world. This situation will increase the consumption of diclofenac sodium [45]. Studies have shown that diclofenac sodium is in the top three most prescribed drugs [46]. Considering all these negative results, it is essential to ensure maximum efficiency of the drug. In order to increase efficiency and not to damage other tissues, it is necessary to ensure the efficient transportation of diclofenac sodium.

## 2. Results and Discussion

In this study, graphene oxide incorporated nanohybrid aerogels (GO-Aero) were produced and characterized, and their drug adsorption and release performance were tested. From the outset of the study, a priority was given to meticulously determining the component concentrations. It was anticipated that the results would provide valuable contributions to the literature and provide advantages in comparative analyses. Furthermore, the presence of similar studies in the literature supports the suitability of this approach. The GO/alginate ratio used in this study is approximately 2:1 (*w*/*v*), which is consistent with the optimum compositions suggested in the literature in terms of both high drug adsorption capacity and controlled release profile. The GO/alginate composition at a ratio of 2:1 offers a balanced and suitable formulation in terms of both drug loading efficiency and extended and controlled release behavior. In the literature, formulations containing GO in the range of 1.15–2% *w*/*w* in GO/alginate hybrid systems have been reported to be effective in terms of physical stability [47]. In other words, the GO/alginate ratio is consistent with the formulations suggested in the literature. This ratio increased the effectiveness of aerogels as pharmaceutical carrier systems by providing both high-efficiency drug loading and sustainable release over time.

The drug adsorption and release performance of aerogel systems depends not only on the type of materials used, but also on the ratios of these materials, preparation conditions, and drying method. While graphene oxide’s surface area and rich structure in terms of functional groups allow it to establish strong interactions with drug molecules, alginate decisively affects the mechanical stability and swelling behavior of the matrix with its hydrophilic and biocompatible structure [48,49]. Drying of aerogels at 70 °C allowed the porous structure to be preserved and a homogeneous structure to be obtained. These findings show that both the composition ratios and the drying method are decisive on the final performance. The ratio of graphene oxide (GO) to alginate is an important factor on the drug adsorption capacity and release kinetics of composite aerogels. GO increases the adsorption capacity by establishing strong π–π, hydrogen bonding, and electrostatic interactions with drug molecules due to its high surface area and various oxygenated functional groups [50]. Therefore, increasing the GO ratio generally positively affects the drug loading efficiency. However, very high GO contents can lead to nanosheet aggregation, reduce the effective surface area, and limit the adsorption [51]. Alginate determines the mechanical strength and swelling behavior of the composite by forming a hydrophilic and biocompatible matrix. High alginate ratios cause the pore structure to expand and the matrix to swell faster in aqueous media, thus allowing the drug to be released faster. The aerogel was dried in a controlled manner using a slow drying method until it reached a porous and structurally stable form. This process minimized pore collapse and preserved the aerogel’s three-dimensional network structure. Furthermore the resulting aerogel can be homogeneously dispersed at the nanoscale within the dispersion medium, offering a significant advantage for both adsorption and release applications.

Alginate is a natural polymer. When used alone as 2D sheets, graphene oxide (GO) tends to stack between the sheets, losing surface area and limiting its adsorption capacity. However, dispersing GO within a three-dimensional alginate-based aerogel matrix allows for both the preservation of GO’s high surface area and the facilitation of diffusion processes thanks to the aerogel’s porous structure. Thus, the 3D structure significantly enhances both drug adsorption capacity and controlled release performance compared to 2D GO sheets.

**Figure 1:**
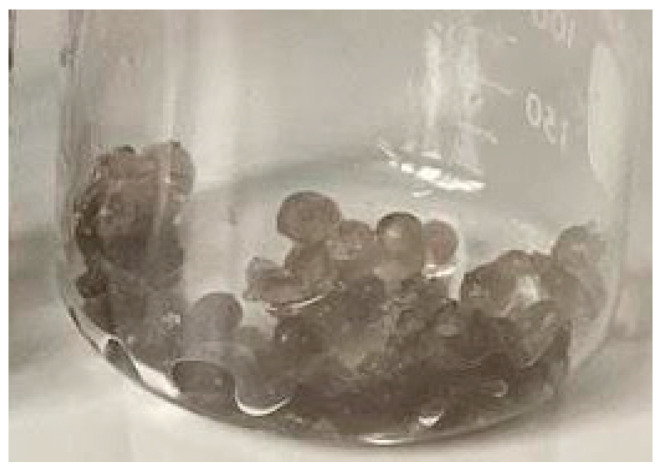
Photograph of the hybrid aerogel beads.

The classical BET method cannot be used to determine the surface area of graphene oxide because it requires a solid sample in powder form. However, when graphene oxide is dried, the layers accumulate and agglomerate, preventing the measurement of the true surface area. Therefore, the methylene blue adsorption method provides more convenient and reliable results for determining the specific surface area of graphene oxide in its dispersed form. Figure 1 shows the photograph of the wet hybrid aerogel beads. The results obtained from the study are given below. The surface area of the produced GO and GO-Aero was calculated using the methylene blue test method. The surface areas were calculated using the results obtained from the methylene blue test and were found to be 597.07 m^2^/g for GO and 489.60 m^2^/g for GO-Aero. Large surface area is an advantage for adsorption. Adsorbent surface area is one of the most important parameters affecting adsorption [52].

**Figure 2:**
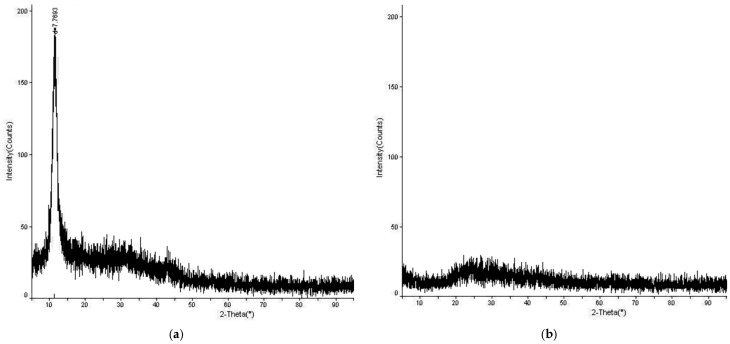
XRD analysis of GO (**a**) and GO-Aero (**b**).

XRD (X-ray diffraction) analysis was performed to elucidate the structure of the produced GO and GO-Aero, initially. The XRD analysis results for GO and GO-Aero are shown in Figure 2a,b. The characteristic peak at 2θ = 11.4° (with an interlayer distance of 0.78 nm) belonging to GO is clearly seen in the XRD pattern of GO given in Figure 2 in accordance with our previous studies [11,27]. The broad peak corresponding to the 2θ value of approximately 25–30° seen in the XRD pattern of GO-Aero given in Figure 2 belongs to the amorphous alginate matrix in the hybrid aerogel structure and shows that GO layers are well dispersed in the alginate matrix. By reason of the fact that the addition of GO did not reveal crystallinity, which is important and related to the uniformity of the hybrid structure, GO peak is not seen in the XRD diagram of the hybrid aerogel [53,54].

**Figure 3:**
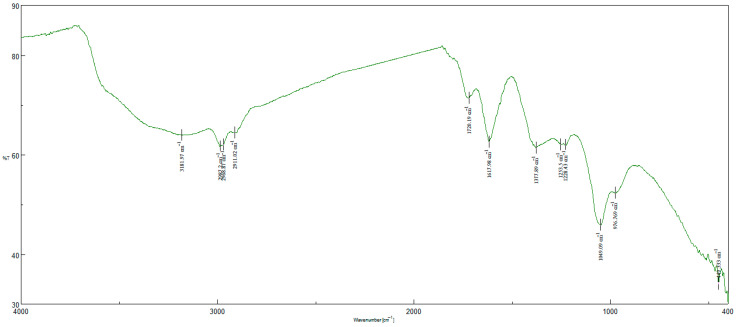
FTIR analysis of GO.

**Figure 4:**
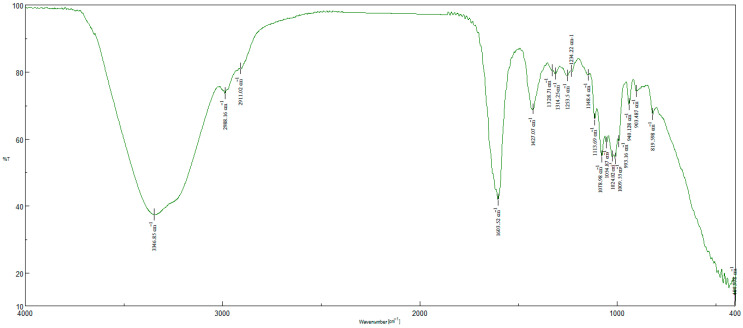
FTIR analysis of GO-Aero.

The FTIR (Fourier Transform InfraRed) analysis results of the produced materials are given in Figure 3 and Figure 4. In the spectra of GO, there are characteristic peaks at around 1000, 1600, and 1700 cm^−1^ which are assigned to the stretching vibrations of C–O, C=C, and C=O, respectively. There is also a characteristic broad band at around 3200 cm^−1^ which is assigned to OH groups. In the spectra of GO-Aero, the peaks at around 1600, 1400, and 3350 cm^−1^ are assigned to the COO^−^ and OH stretching vibrations. The small peaks at around 2950 cm^−1^ can be attributed to the C–H stretching vibrations [55,56,57,58]. FTIR analysis results showed that GO and GO-Aero were synthesized successfully along with the XRD diagrams.

**Figure 5:**
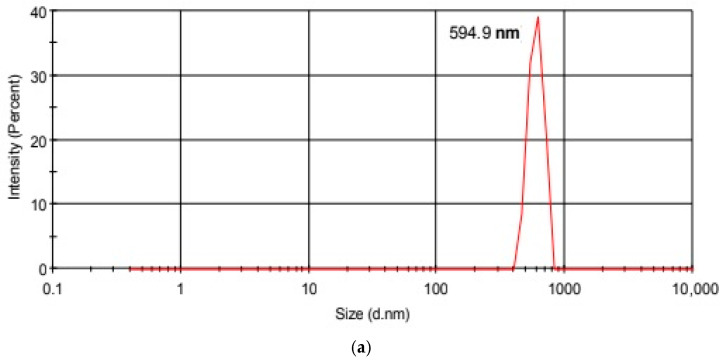

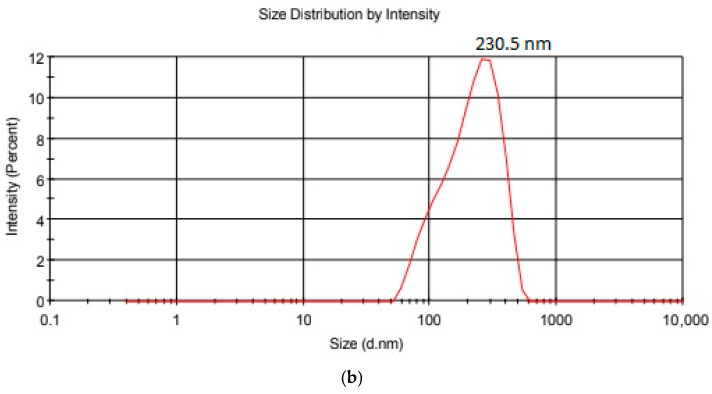
Particle size distribution of GO (**a**) and GO-Aero (**b**).

Figure 5 shows the particle size distribution of GO and GO-Aero as the result of dynamic light scattering (DLS) analysis. GO particles have a hydrodynamic size of around ~590 nm according to DLS analysis results given in Table 1. Figure 5a also shows a sharp peak indicating the uniformity in particle size distribution with a reasonable PdI value which is 0.676. Particle size distribution of GO-Aero shows a broad peak which has a maximum of ~230 nm with a Polydispersity Index (PDI) value of 0.461. Particle sizes of the final product (GO-Aero) are smaller than GO with a good PDI value. This observed result showed that the produced material secured the GO layers to disperse better in the dispersion medium and prevented them from aggregation. Similar particle size values were reported for alginate-based composite nanostructures in the literature [59].

**Figure 6:**
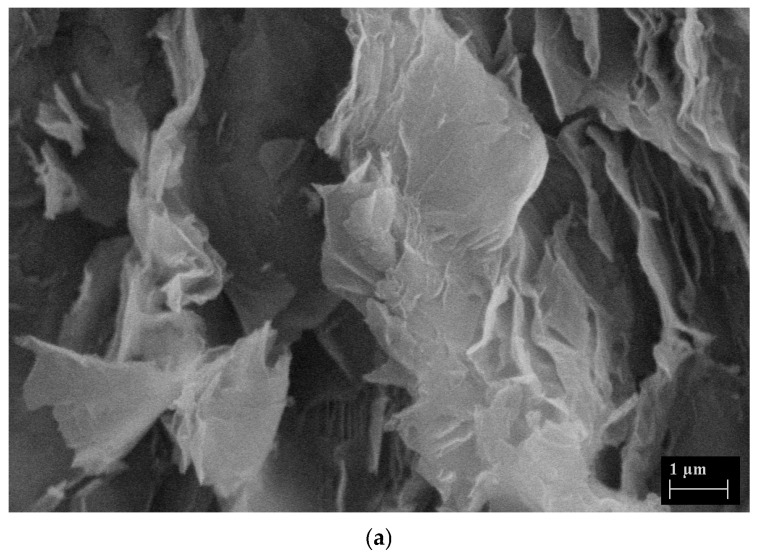

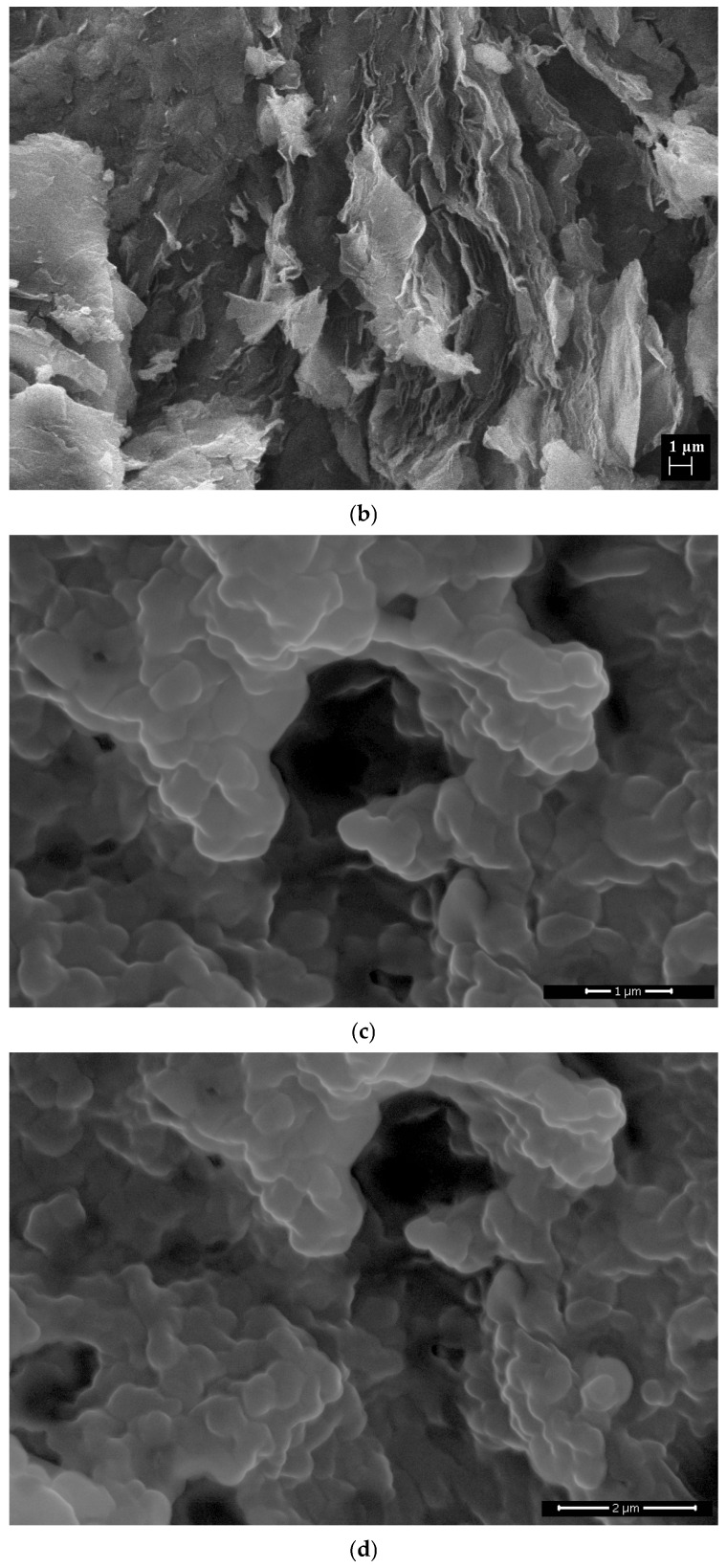
SEM images of GO (**a**,**b**) and GO-Aero (**c**,**d**).

**Figure 7:**
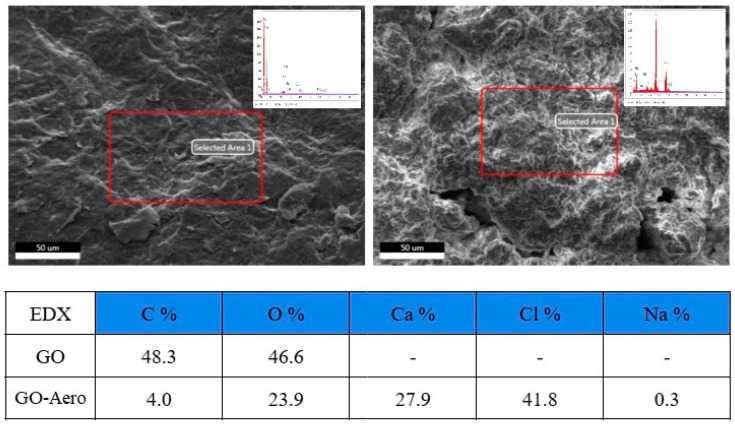
EDX analysis results of GO (**Left**) and GO-Aero (**Right**).

The produced GO and the nanohybrid aerogels were scanned using SEM (Scanning Electron Microscope) and the SEM photographs are given in Figure 6. Figure 6a,b show the layered structure of GO and Figure 6c,d show the porous structure and inhomogeneous surface of the nanohybrid aerogels (GO-Aero), encapsulated by the graphene layers inside and well dispersed. EDX analysis was also performed to make a semiquantitative evaluation of the chemical composition of the produced materials (GO and GO-Aero), demonstrated in Figure 7. Energy Dispersive X-ray (EDX) analysis results show the main components of GO which are carbon and oxygen with an approximate C/O ratio of 1.04 showing the successful oxidation of graphite during GO production [60]. In the case of GO-Aero, the sample also contains Ca, Cl, Na as expected for the alginate structure crosslinked with Ca ions, as well as C and O [61].

**Figure 8:**
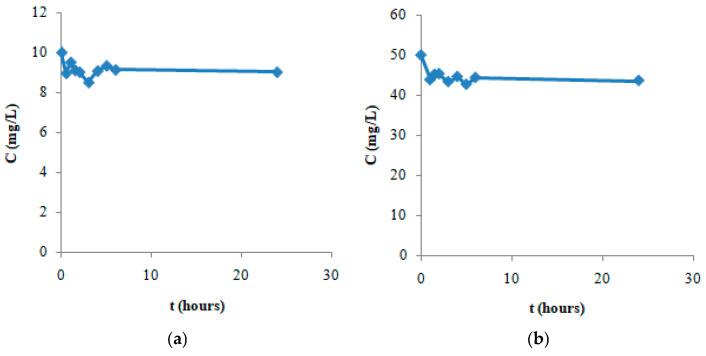
Graphs of the change in the concentration of DS adsorption on GO-Aero with time for the initial DS concentration of 10 mg/L (**a**) and 50 mg/L (**b**).

**Figure 9:**
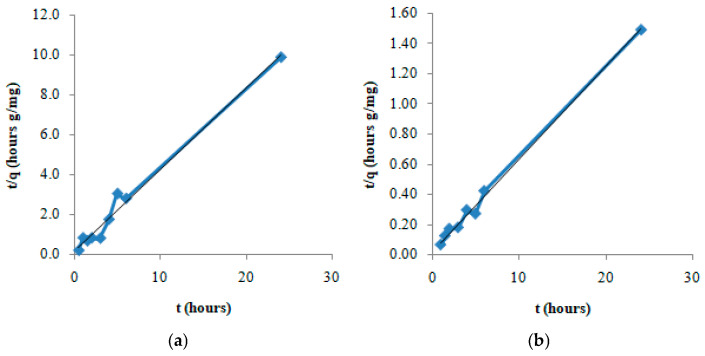
Pseudo-2nd order graphs of the adsorption of DS on GO-Aero for the initial DS concentration of 10 mg/L (**a**) and 50 mg/L (**b**).

After the characterization studies, the adsorption of the selected model drug DS on GO-Aero was investigated and the results obtained are given below. First of all, samples were taken from the adsorption medium at predetermined time intervals and the progress of the adsorption process was monitored over time. The results of the experiments conducted using initial concentrations of DS which are 10 mg/L (Figure 8a) and 50 mg/L (Figure 8b) are given in Figure 8. According to the results obtained, the equilibrium time for the adsorption of DS on GO-Aero was found to be 3 h. Adsorption kinetics were modeled using the obtained concentration change data with time. It was determined that DS adsorption did not fit the Lagergren 1st order model (Equation (5)) but fitted the Pseudo-2nd order model (Equation (6)) according to correlation coefficient (R^2^) values. The Pseudo-2nd order graphs are shown in Figure 9, and the kinetic data of the adsorption were calculated and shown in Table 2. Adsorption of DS on GO-Aero was completed practically in 3 h and followed the Pseudo-2nd order kinetic model similar to our previous reports on the adsorption of drug molecules on various adsorbents [62,63].

**Figure 10:**
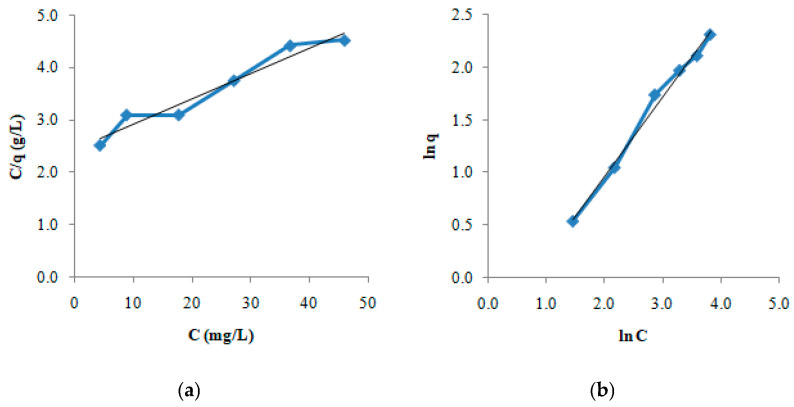
Langmuir (**a**) and Freundlich (**b**) isotherm graphs of the adsorption of DS on GO-Aero.

**Figure 11:**
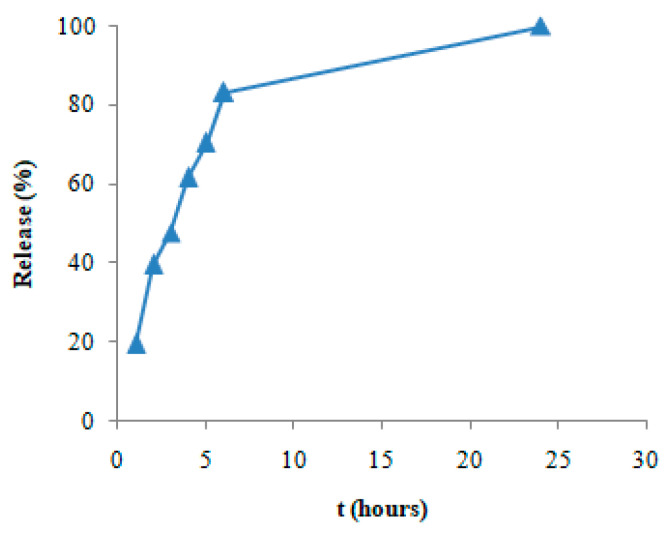
In vitro release profile of DS.

The adsorption of DS on GO-Aero was investigated in the concentration range of 10–50 mg/L using the found equilibrium time which is 3 h. The obtained data were modeled using the Langmuir (Equation (3)) and the Freundlich (Equation (4)) isotherms. The graphs of the models are shown in Figure 10a,b and the isotherm constants calculated are given in Table 3. As seen in Table 3, the adsorption capacity of GO-Aero for DS was found to be 20.83 mg/g. Isotherm data also showed that the adsorption process complied with the Langmuir and Freundlich equations with high correlation coefficient (R^2^) values. After proving the adsorption capacity, in vitro release of DS was also investigated over 24 h and the result is given in Figure 11. In vitro release profile of DS shows the slow and gradual release of DS during the time after the burst release effect in the initial hours which is favorable for drug carrier systems [64]. The results obtained from the adsorption and release studies revealed that the produced graphene incorporated hybrid aerogel (GO-Aero) could be a potential drug adsorbent.

DS has a pKa value of 4.0 and is present as its anionic form in the aqueous medium at pH values higher than 4.0 [65], which is the case of the GO-Aero dispersion that has a natural pH of 6.2 (Table 1). The GO-Aero surface is also negatively charged according to its zeta potential value at the same pH which is −22.6 mV (Table 1). It is seen that there is an electrostatic repulsion between the drug molecules and the surface of the nanohybrid aerogels. Equilibrium data, where 1/n (0.76) < 1, indicates that the adsorption process is favorable (Table 3). Taking into account all the given information above, DS adsorption on GO-Aero can be explained by the π–π dispersion interactions along with a possible hydrophobic interaction as previously reported in our studies for the adsorption of drug molecules on carbon-based and organo-modified adsorbents [62,63,66,67].

Adsorption studies of DS on GO-Aero are important parts of the controlled drug delivery process. In previous adsorption studies on the controlled delivery of DS, the potential of MIL-88A metal–organic frameworks with different morphologies and varying surface areas of 326 and 307 m^2^/g in the targeted transport of DS in migraine treatment was demonstrated. While the highest adsorption capacity due to the effect of hydrogen bonding and electrostatic interactions was attributed to MIL-88a-1, when drug release was examined, MIL-88A carriers showed drug release in the range of 58–97% in 24 h at pH 6.8 [68]. The extent to which sodium alginate/carbon films prepared with calcium chloride absorb DS was investigated by analyzing various operating factors such as 1 g/L adsorbent, initial DS concentration ranging from 10 to 50 mg/L similar to our study, different adsorption temperatures (303 to 343 K), and different pH (3 to 11). The porous and irregular structure of alginate/carbon films with a surface area of 35.16 m^2^/g was attributed to activated carbon. When the adsorption capacity was considered, the best maximum adsorption capacity (Q) value was found to be 29.898 mg/g at 50 mg/L DS concentration, pH 3, and 303 K [69].

Double-layer adsorption of DS onto luminescent nanoparticles at different temperatures (25, 37 °C) and pHs (7.4, 5.2) and the controlled transport and therapeutic effects of the drug in the inflammatory environment were determined. Luminescent nanoparticles are agents that carry and release different types of molecules. In the experiment conducted on 2 mg adsorbent (cAp and cAp-Tb) at various concentrations of DS ranging from 0.05 to 0.45 mg/mL with both neat and luminescent Terbium 3+ (Tb 3+) citrate coated carbonated apatite nanoparticles (cAp), it was observed that both agents were effective in adsorption and the adsorption capacity of cAp-Tb on DS was higher than cAp without Tb. The highest adsorption of DS onto cAp-Tb nanoparticles was found to be 6.3606 mg/g at 37 °C, followed by 5.411 mg/g adsorption capacity at 25 °C. It was reported that this was an indication of higher affinity and cooperativity. Successful drug delivery due to therapeutic effect was reported with successful adsorption [70]. When the adsorption of DS on granular activated charcoal was examined, the adsorption capacity was found to be 14.73 mg/g [71]. For 10 mL of DS solution prepared at pH 4.5, the adsorption capacity was determined on double-layered amino-functionalized cellulose nanocrystals/chitosan composites. The adsorption capacity was found to be 45.11 mg/g for cellulose nanocrystals at 100 mg/L DS concentration, while it was 45.97 mg/g for chitosan composites [72]. DS adsorption was performed on reduced graphene oxides with monolayer loading. It was observed that the maximum adsorption capacity was 59.67 mg/g with 200 min adsorption time [73]. Polyethyleneimine functionalized sodium alginate/cellulose nanocrystals/polyvinyl alcohol core–shell microspheres were produced for the adsorption of DS. In the study carried out at different temperatures such as 291, 297, and 303 K for 50 min adsorption time at 4.5 pH, it was observed that the maximum adsorption capacity was positively correlated with temperature [72].

In another study investigating the adsorption capacity of DS onto non-homogeneous, porous GO@CoFe_2_O_4_ nanocomposite with a surface area of 239.5 m^2^/g, DS solutions were used at different concentrations (1.6–18.4 mg/L) and pH values (2.6–9.4). The maximum adsorption capacity was found to be 32.37 mg/g. High compatibility with the Pseudo-second order model was observed (R^2^ ≥ 0.9970). According to Langmuir isotherm data, the maximum adsorption capacity (Q) was 18.4 mg/g at 10.5 mg/L drug concentration and pH 4 on the structure without graphene oxide (CoFe_2_O_4_). The addition of graphene oxide increased the maximum adsorption capacity [74]. In adsorption experiments with DS solutions at concentrations ranging from 50 to 125 mg/L, nanocomposite hydrogel (Fe_3_O_4_@SiO@APTMS(3-Aminopropyl trimethoxysilane)@MAN (Maleic anhydride) hydrogel (MNPsH)) was used as an adsorbent. With the results showing high compliance with the Langmuir isotherm, maximum adsorption capacity (Q) of 28.92, 28.11, and 29.27 mg/g was found for hydrogels with different weights of 0.6, 0.75, and 0.9 g, respectively. When drug release potential was examined, cumulative release of up to 93% was observed within 120 min at pH 7.4 [75].

Carbon nanotubes (CNT’s) with a surface area of 132.99 m^2^ g^−1^ were composited with different gelatins and bead composites were obtained. DS loading was applied to these gelatin bead composites with different swelling capacities. According to Langmuir isotherm data, the maximum adsorption capacity was found to be 26.97 mg/g in commercial gelatin/CNT beads, while the value was found to be 20.57 mg/g in RCTLW (recovered from chromium-tanned leather waste) gelatin/CNT beads produced from waste. However, according to Langmuir isotherm, Pseudo-first order (Lagergren) and pseudo-second order data, R^2^ is higher in RCTLW gelatin/CNT beads, meaning compatibility is high [76]. The adsorption capacity of phyllosides modified with octadecyldimethylbenzylammonium chloride (O) and dodecylamine (D) surfactants for DS at different initial concentrations and different times was tested. The maximum adsorption capacities for O and D composites were found between 12.3 and 38.4 mg/g depending on the increase in the initial concentration of the drug. The adsorption capacity increased as the initial concentration increased. The highest value was observed in octadecyldimethylbenzylammonium chloride. Similar chain length ensures strong adsorption [77]. When the studies in the literature are examined, adsorption studies for the targeted distribution of DS are insufficient. The aerogel produced in our study is the first in the literature in terms of adsorption and controlled release of DS and will shed light on future studies.

In this study, DS adsorption performance of graphene oxide (GO)-based nanohybrid aerogels developed was investigated in detail, and the maximum adsorption capacity was determined as 20.83 mg/g according to the Langmuir isotherm. The achievement of adsorption equilibrium in a short time of 3 h demonstrates the material’s rapid interaction capacity. Graphene oxide has a layered structure and the layers tend to stack on top of each other. However, the homogeneous distribution of graphene oxide within the aerogel matrix enables the efficient use of its large surface area for drug loading. In this way, the high surface area advantage of graphene oxide is successfully combined with the porous structure of the aerogel matrix. Furthermore, the slow drying method at ambient pressure [78] significantly contributes to the preservation of the porous structure. Although systems with higher adsorption capacities have been reported in the literature (e.g., 596.71 mg/g for 3D rGO aerogel [79]; 605.87 mg/g for CNC–PVAm/rGO [80]), the priority in this study was to develop a controlled, stable, and biocompatible release system rather than a high-dose loading capacity. In this context, the developed nanohybrid aerogel is considered a suitable candidate for low-dose, long-lasting pharmaceutical delivery systems. With its stable dispersion properties, controlled kinetics, and structure close to physiological pH, this material offers a functional platform for future drug delivery technologies.

## 3. Conclusions

Drug delivery requires material innovation. Positive results can be obtained by using various materials in the targeted delivery of diclofenac sodium (DS). In our study, monolayer loading of diclofenac sodium onto a new material for controlled drug delivery was performed. The graphene oxide nanohybrid aerogels developed in this study offer significant advantages in terms of environmental sustainability. It is known that common pharmaceutical pollutants such as diclofenac accumulate in surface waters and cause toxic effects on aquatic organisms. Since such compounds cannot be sufficiently removed by conventional wastewater treatment systems, a controlled and effective intervention in the environment is required [81]. The developed aerogels offer an effective alternative for the adsorption of these pollutants thanks to their high surface area and functional structures, while at the same time helping to prevent the random spread of pharmaceutical substances into the environment with their controlled drug release capacity. Biopolymers such as sodium alginate used in the synthesis of aerogels comply with the principles of green chemistry with their renewable source, biodegradable, and non-toxic structures. In addition, working with water-based solvents and production with low energy requirements are important in terms of reducing the carbon footprint. The reusability of the material coincides with the circular economy goals by reducing waste production and raw material consumption. In this context, the developed nanohybrid aerogels stand out as versatile materials that contribute not only to the removal of pharmaceutical pollutants but also to sustainable environmental technologies. A new aerogel consisting of sodium alginate and graphene oxide was produced as a drug adsorbent. In our study, FTIR analysis was performed to obtain data on the structure of the synthesized adsorbent. Before loading, it was proven by FTIR that graphene oxide and nanohybrid aerogel were successfully produced. It was observed that the adsorption capacity of the nanohybrid aerogels we produced was not affected by different temperatures and pH conditions. It was observed that the change in the amount of adsorbed substance over time was completed in three hours (Figure 8a,b). In our study, it was found that the surface area of the produced nanohybrid aerogel is 489.60 m^2^/g. Since the porous structure of the adsorbent will facilitate the adhesion of molecules or atoms to be adsorbed, the amount of adsorption will increase as the adsorbent porosity increases. The porous and inhomogeneous structure in the SEM images in Figure 6 is an indication that the adsorbent was produced successfully. The obtained data fit the Pseudo-second order rate equation and kinetic parameters affecting adsorption such as the rate constants (k_2_) were calculated. Depending on the concentration of the adsorbate, it was observed that the adsorption quantity at equilibrium increased with the increase in the concentration of DS, while the rate constant k_2_ decreased with the increase in the concentration of DS. By applying the obtained data to the equations of the Langmuir and the Freundlich isotherms and transferring them to their graphs, it was seen that the adsorption character was suitable for both models. In the Freundlich isotherm data, 1/n (0.76) < 1 indicates that the process is favorable. There are π–π dispersion interactions between the adsorbent and the adsorbate. The results obtained from the study is that the equilibrium time of the adsorption of DS on composite aerogel is 3 h and the maximum adsorption capacity of the adsorbent is 20.83 mg/g. It is thought that the results obtained from this study will provide information for drug production and will be useful for future studies on the improvement of existing treatments.

## 4. Materials and Methods

Graphite, sulfuric acid, phosphoric acid, potassium permanganate, hydrogen peroxide, hydrochloric acid, diclofenac sodium, sodium alginate, and calcium chloride were supplied by Sigma (Goldbach, Germany) in analytical grade. All the chemicals used were the highest purity available and used as received without any additional purification.

### 4.1. Production of the Graphene Oxide Incorporated Hybrid Aerogels

**Figure 12:**
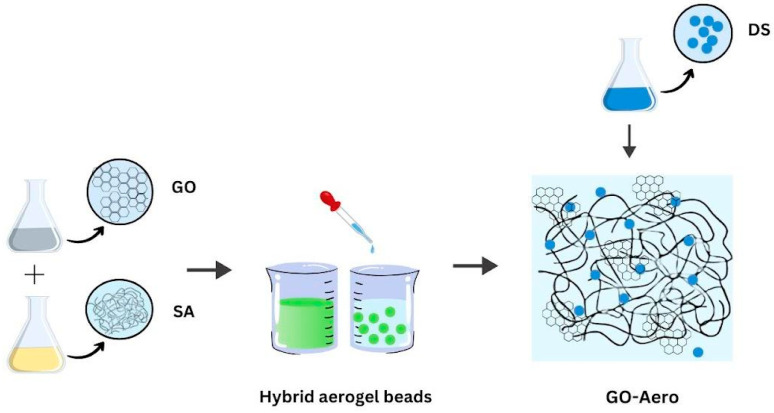
Production method of GO incorporated nanohybrid aerogels.

Graphene oxide (GO) was produced by using the Hummers method [6] modified in our laboratory. Powdered graphite, sulfuric acid (H_2_SO_4_), phosphoric acid (H_3_PO_4_), potassium permanganate (KMnO_4_), and hydrogen peroxide (H_2_O_2_) were used for GO synthesis. In this method, sulfuric acid and phosphoric acid are mixed in a beaker and graphite added in parts. Potassium permanganate is slowly added to this mixture in the same way. The resulting dark green dispersion is continued to be stirred with a magnetic stirrer. Then, this dispersion is stopped mixing and is left for three days. On the fourth day, hydrogen peroxide is dropped onto this mixture with a dropper and a bright yellow color is observed. The synthesized GO is first washed with 5% hydrochloric acid (HCl) solution and then with pure water until a neutral pH value is observed. The GO separated by centrifugation is dried at 85 °C. The aqueous dispersions of the produced GO (4% *w*/*v*) were used in the production of graphene-based aerogels. GO aerogels were produced in the form of beads by adding sodium alginate solution (2% *w*/*v*) to graphene oxide dispersions in water and then dropping them into calcium chloride (CaCl_2_) solution (3% *w*/*v*). The produced aerogels were washed with pure water and dried first in air and then at 70 °C. The production method is summarized schematically in Figure 12.

### 4.2. Characterization of the Nanohybrid Aerogels

Within the scope of the study, samples were taken from the produced materials and their physicochemical characterization was carried out. Characterization of the materials was carried out using X-ray diffraction (XRD) analysis, Fourier Transform InfraRed (FTIR) Spectrophotometer, Zetasizer Instrument, and Electron microscope (SEM-EDX). Methylene blue test was applied for surface area determination and the obtained results are given under Section 2.

### 4.3. Investigation of Drug Adsorption on Nanohybrid Aerogels

The loading of the selected model drug, diclofenac sodium (DS), onto the nanohybrid aerogels was carried out using the in vitro batch adsorption method at 25 °C. Drug solutions of appropriate concentration were mixed with graphene-based aerogel dispersions, samples were taken at predetermined time intervals and absorbance measurements were made using a UV (ultraviolet)–Visible spectrophotometer. In the spectrophotometric method based on measuring the absorbance of molecules in the UV or visible areas, the equation of the Lambert–Beer law (1) given below was used to calculate the concentration.

A = εCl(1)

In addition, the effect of different temperatures and pH on this adsorption was investigated and it was determined that they did not have a significant effect on the adsorption capacity (plots are not given) of DS on the produced aerogels [66]. As a result of studies conducted with different experimental periods, it was determined that the time for the adsorption of DS on the produced aerogels to reach equilibrium was 3 h. Adsorption kinetics were modeled using Lagergren first (5) and Pseudo-second order (6) rate equations. Adsorption capacities were found by drawing Langmuir (3) and Freundlich (4) adsorption isotherms. Each experiment was repeated at least three times. The amount of adsorbed substance was calculated by taking the difference in the adsorbate concentration in the medium before and after adsorption. The following Equation (2) was used:

(2) $$q=\frac{{(C}_{0}-C)}{m}V$$

The linear form of the Langmuir isotherm is as follows:

(3) $$\frac{C_{e}}{q_{e}}=\frac{1}{Qb}+\frac{C_{e}}{Q}$$

The constants Q and b, which provide information about the adsorption capacity of the adsorbent, are calculated from the slope and intercept of the graph drawn between C_e_/q_e_ and C_e_.

Freundlich isotherm was derived based on experimental studies for adsorptions on solid surfaces that are not ideally clean and homogeneous (as considered in the derivation of the Langmuir equation). The linear form of the Freundlich isotherm is as follows:

(4) $$lnq_{e}=lnk_{F}+\frac{1}{n}lnC_{e}$$

where 1/n is found from the slope of the graph drawn between lnq_e_ and lnC_e_, and k_F_ is found from the point where the curve intersects the ordinate.

Adsorption kinetics were modeled using the linear form of the Lagergren 1st order rate Equation (5) and the Pseudo-2nd order rate Equation (6) given below, respectively.

*l*n(q_e_ − q) = *l*nq_e_ − k_1_t (5)

A line is obtained when a graph is drawn between ln(q_e_ − q) and t. The adsorption rate constant, k_1_, is found from the slope of the line.

The linear form of the Pseudo-2nd order rate equation is as follows.

(6) $$\;\frac{t}{q}=\frac{1}{k_{2}q_{e}^{2}}+\frac{t}{q_{e}}$$

A line is obtained when a graph is drawn between t/q and t. The adsorption rate constant, k_2_, is found from the slope and intercept of the line.

### 4.4. In Vitro Release Study of the Nanohybrid Aerogels

In vitro release of DS from the nanohybrid aerogels was studied spectrophotometrically (Shimadzu 2100S) using dialysis bags in a PBS (phosphate-buffered saline, pH 7.4) medium. 25 mg of drug loaded nanohybrid aerogel and 50 mL of PBS were used for the release experiments. Release experiments were conducted in a thermostatic shaking water bath at 37 °C. Samples were taken at predetermined time intervals (at 1 h, 2 h, 3 h, 4 h, 5 h, 6 h, and 24 h) and the concentration of the samples were calculated by spectrophotometric method using the calibration curves prepared initially [82].

## Figures and Tables

**Table 1:** Hydrodynamic particle size, poly-dispersity index (PDI) and zeta potentials of GO and GO-Aero.

	Hydrodynamic Particle Size (nm)	PDI	Zeta Potential	pH of the Dispersion
GO	594.9	0.676	−4.20	5.6
GO-Aero	230.5	0.461	−22.6	6.2

**Table 2:** Kinetic parameters for the adsorption of DS on GO-Aero.

	Pseudo 2nd Order Model			Lagergren 1st Order Model		
Concentration (for DS)	k_2_	q_e_	R^2^	k_1_	q_e_	R^2^
10 mg/L	0.967	2.44	0.983	-	-	0.347
50 mg/L	0.186	16.39	0.995	-	-	0.077

**Table 3:** Isotherm data for the adsorption of DS on GO-Aero.

	Langmuir Isotherm			Freundlich Isotherm		
	Q (mg/g)	b (L/g)	R^2^	1/n	k_F_	R^2^
DS	20.83	0.02	0.946	0.76	0.58	0.990

## Data Availability

The data that support the findings of this study are available from the corresponding author, [E.C.S.], upon reasonable request.

## Abbreviations

The following abbreviations are used in this manuscript:

GO	Graphene oxide
RGO	Reduced graphene oxide
XRD	X-ray diffraction
FTIR	Fourier transform InfraRed
PDI	Polydispersity index
SEM	Scanning Electron Microscope
EDX	Energy Dispersive X-ray analysis
DS	Diclofenac sodium
CNT	Carbon nanotubes
DLS	Dynamic light scattering
